# 

**Published:** 1906-03

**Authors:** 


Please mention this Journal.

MAR. 16 1906

SUBSCRIPTION $1.00.

PUBLISHED MONTHLY.

### ATLANTA JOURNAL-RECORD OF MEDICINE.

Successor to Atlanta Medical and Surgical Journal, Established 1855, and Southern Medical Record, Established 1870.

VOL. VII. ATLANTA, GA., MARCH, 1906. No. 12.

